# Visit‑to‑visit variability of inflammation–immunity indices and prognosis in hepatocellular carcinoma

**DOI:** 10.1186/s12876-025-04506-6

**Published:** 2025-12-24

**Authors:** Qiajun Du, Youli Zhao, Jing Yang, Yongxin Yang

**Affiliations:** Laboratory Medicine Center, The Second Hospital & Clinical Medical School, Lanzhou University, 82 Cuiyingmen, Lanzhou, Gansu 730030 China

**Keywords:** Hepatocellular carcinoma, Systemic immune-inflammation index, Visit-to-visit variability, Overall survival, Prognostic modeling

## Abstract

**Background:**

Inflammatory–immune blood indices such as the systemic immune‑inflammation index (SII) relate to outcomes in hepatocellular carcinoma (HCC). Visit‑to‑visit variability (VVV) may capture instability of host inflammatory homeostasis that is not reflected by average levels. We examined whether early VVV of these indices predicts overall survival (OS) and adds discriminatory value beyond standard prognostic factors and mean levels.

**Methods:**

We conducted a single‑center retrospective cohort of adults with HCC from June 2020 to December 2024. All laboratory “visits” within days 0–90 informed per‑patient VVV; the primary exposure was SII variability independent of the mean (SII‑VIM), with average real variability (ARV) and SD/CV% as secondary metrics. The primary outcome was OS from the day‑90 landmark. Predictive increment was assessed by change in Harrell’s C‑index, time‑dependent AUC (1 and 2 years), and NRI/IDI with bootstrap internal validation; sensitivity analyses included a 0–180‑day window, time‑varying rolling VVV, and multiple imputation (m = 20).

**Results:**

Of 780 patients, 528 survived to the landmark and were analyzed. Median follow-up was 25.1 months with 216 deaths (41%). Each 1-SD increase in SII-VIM was associated with higher mortality (adjusted HR 1.26, 95% CI 1.12–1.41); the highest versus lowest quartile yielded HR 1.72 (95% CI 1.25–2.36). Splines showed a monotonic dose–response. Adding SII-VIM improved discrimination from C-index 0.68 to 0.71 and increased AUC from 0.70 to 0.73 at 1 year and from 0.67 to 0.70 at 2 years; continuous NRI was 0.14 (95% CI 0.06–0.22) and IDI 0.018 (95% CI 0.008–0.030). Results were consistent using ARV (HR 1.20), a 0–180-day window (HR 1.22), and time-varying analyses (HR 1.18).

**Conclusions:**

Early visit-to-visit variability—particularly SII-VIM—was independently associated with worse OS in HCC and added modest, clinically meaningful discrimination beyond standard factors and mean levels, and prospective validation is warranted.

**Supplementary Information:**

The online version contains supplementary material available at 10.1186/s12876-025-04506-6.

## Introduction

 Hepatocellular carcinoma (HCC) remains a significant global health challenge, ranking as the third leading cause of cancer-related mortality worldwide [1–3]. The incidence of HCC is notably higher in Asia and Africa compared to Europe and North America [2]. Despite the existence of established staging systems like the Barcelona Clinic Liver Cancer (BCLC) and the Hong Kong Liver Cancer systems, these frameworks often fall short in addressing the heterogeneity of HCC, which is influenced by tumor burden, liver function, and patient performance status [1, 4, 5]. The BCLC system, while widely recognized, has limitations, particularly in its handling of intermediate and advanced stages [5, 6]. Prognostic tools that incorporate liver function assessments, such as the ALBI and Child-Pugh scores, are crucial but insufficient for individualized risk stratification [6, 7]. Advances in biomarker discovery, including the use of alpha-fetoprotein (AFP), glypican 3 (GPC3), and des gamma carboxy prothrombin (DCP), alongside emerging technologies like next-generation sequencing and liquid biopsies, offer promising avenues for improving HCC management [7, 8]. However, challenges remain in validating these biomarkers and integrating them into clinical practice to enhance personalized treatment approaches and ultimately improve patient outcomes.

Peripheral blood indices such as the systemic immune-inflammation index (SII), neutrophil-to-lymphocyte ratio (NLR), platelet-to-lymphocyte ratio (PLR), lymphocyte-to-monocyte ratio (LMR), systemic inflammation response index (SIRI), prognostic nutritional index (PNI), and albumin-bilirubin index (ALBI) have been identified as significant prognostic markers for various cancers, including HCC, colorectal cancer, oral cancer, and breast cancer. These indices are derived from routine blood tests and reflect the balance between pro-inflammatory and anti-inflammatory responses, which are crucial in cancer progression and patient outcomes. For instance, high SII and NLR are associated with poor overall survival (OS) and disease-free survival (DFS) in HCC [9–11]. Similarly, high SII and SIRI are linked to worse OS and DFS in oral and breast cancers [12, 13]. These indices are valuable for stratifying patients based on their risk and guiding treatment decisions, as they are accessible, reliable, and cost-effective [14]. However, the prognostic utility of these indices is often limited by their assessment at a single time-point, which can be affected by temporal instability and sensitivity to intercurrent events such as infections or other inflammatory conditions. This limitation can lead to regression to the mean, where extreme values tend to move towards the average upon repeated measurements, potentially affecting the accuracy of these indices as prognostic tools [14]. Therefore, while these indices provide valuable insights into patient prognosis, their predictive performance could be enhanced by considering dynamic changes over time rather than relying solely on baseline measurements [15, 16].

Visit-to-visit variability (VVV) encapsulates fluctuations in biological measurements over time, potentially indicating instability in inflammatory-immune homeostasis. This concept, previously established in cardiometabolic research, highlights that high VVV in parameters such as blood pressure, glucose, and cholesterol correlates with increased risks of cardiovascular disease and mortality [17–19]. In HCC, biological plausibility arises from the oscillations in neutrophil and platelet-driven inflammation alongside lymphocyte-mediated immunity, which can be affected by factors like infection and treatment responses [20]. The dynamic nature of VVV suggests that monitoring these fluctuations could provide critical insights into patient outcomes and disease progression in HCC [17].

Because single time‑point indices can be misleading and raw variability can be dominated by average level, we used VIM to capture instability that is independent of the mean, an approach with precedents in other clinical domains. We investigated whether early visit‑to‑visit variability in inflammation–immunity indices—particularly SII‑VIM—adds independent and incremental prognostic information for OS in HCC. We hypothesized that higher SII‑VIM over days 0–90 (day‑90 landmark) would be associated with worse OS after adjustment for established clinical factors, the patient‑specific mean SII, and testing frequency. Secondary aims included extending VVV to NLR, PLR, LMR, SIRI, PNI, and ALBI; robustness checks (0–180‑day window with a day‑180 landmark; time‑varying rolling updates); and assessment of incremental discrimination and reclassification (ΔC‑index, time‑dependent AUC at prespecified horizons, continuous NRI/IDI) with internal validation and FDR control.

## Method

### Study design

We conducted a single-center, retrospective cohort study of adults with hepatocellular carcinoma (HCC) that was approved by the institutional ethics committee of The Second Hospital & Clinical Medical School, Lanzhou University with a waiver of informed consent for de-identified data.

### Participants

Eligible patients were ≥ 18 years old, met standard diagnostic criteria for HCC, initiated first-line therapy at our institution, and had at least three same-day laboratory “visits” within 0–90 days from the index date. We excluded patients with another active malignancy, those who died or were lost to follow-up before day 90, and those with extreme acute events during the exposure window that could grossly distort blood counts (major bleeding requiring transfusion or major surgery).

### Index date, landmark, and follow-up

The index date was the calendar day of initiating first-line therapy (curative resection/ablation, locoregional therapy, or systemic therapy). To mitigate immortal-time bias, we defined a day-90 landmark; variability exposures were computed using all laboratory visits from day 0 through day 90, and only patients alive at the landmark were included for time-to-event analyses.

### Laboratory measurements and inflammation–immunity indices

For each laboratory visit, we required same-day complete blood count and chemistry profiles. When multiple draws occurred on the same day, values were averaged to yield a single visit-level measurement. Indices were computed using SI units as follows: systemic immune-inflammation index (SII) = platelets × neutrophils/lymphocytes; Neutrophil-to-lymphocyte ratio (NLR) = neutrophils/lymphocytes; platelet-to-lymphocyte ratio (PLR) = platelets/lymphocytes; lymphocyte-to-monocyte ratio (LMR) = lymphocytes/monocytes; systemic inflammation response index (SIRI) = neutrophils × monocytes/lymphocytes; prognostic nutritional index (PNI) = albumin (g/L) + 5 × lymphocytes (×10⁹/L); and ALBI score = 0.66×log₁₀(bilirubin, µmol/L) − 0.085×albumin (g/L).

### Quantification of visit-to-visit variability (VVV)

Patients with higher average values of a biomarker often show larger ups‑and‑downs simply because the scale is bigger; a raw standard deviation would therefore confound “how much it swings” with “how high the average is.” VIM corrects for this by rescaling the SD by the mean raised to a data‑derived exponent β (VIM = 100 × SD/mean^β). If β were exactly 1, VIM would equal the familiar coefficient of variation; estimating β from the cohort lets us tailor the correction to the observed mean–variance relationship for SII. In practical terms, larger VIM = more instability than expected given the person’s average level.

The primary exposure was variability independent of the mean (VIM) for SII, calculated per patient as 100 × SD/mean^β, where SD and mean refer to all SII values within days 0–90 and β is the slope from regression of ln(SD) on ln(mean) across the cohort. We estimated the cohort‑wide scaling exponent β by ordinary least squares regression of ln(SD) on ln(mean) across patients within the 0–90‑day window and computed VIM = 100 × SD/mean^β for each patient. To assess potential subgroup heterogeneity in the mean–variance relationship, we additionally fit models with interaction terms: ln(SD) ~ ln(mean) × subgroup (pre‑specified: BCLC stage; secondary: therapy class, ALBI grade). From these, we obtained subgroup‑specific β estimates (β_g) and recomputed SII‑VIM(β_g) as a sensitivity analysis. We report the cohort‑wide β (95% CI), Wald tests for slope heterogeneity, the correlation between SII‑VIM(cohort‑β) and SII‑VIM(β_g), and the OS association when using SII‑VIM(β_g). We also derived average real variability (ARV) as the mean of absolute successive differences, SD, and coefficient of variation (CV% = 100 × SD/mean) for sensitivity analyses. All VVV metrics were Z-standardized (mean 0, SD 1) prior to modeling to enable hazard ratios (HRs) per 1-SD increase. For descriptive and triage purposes, we prespecified “high variability” as SII‑VIM ≥ the cohort’s 75th percentile (Q4 boundary) on the raw scale (prior to Z‑standardization). Where only standardized values are available, SII‑VIM Z ≥ + 1 SD can serve as an alternative flag.

### Outcomes

The primary outcome was OS from the day-90 landmark. Secondary outcomes were recurrence-free survival (RFS) in the curative-therapy subset and progression-free survival (PFS) in the non-curative subset; RFS considered death as a competing event using Fine–Gray subdistribution hazards, whereas PFS used a Cox model in counting-process form.

### Covariates

Multivariable models adjusted for prespecified covariates based on subject-matter knowledge and directed acyclic graph considerations: age, sex, BCLC stage (A/B/C), ALBI grade, alpha-fetoprotein (AFP, coded as continuous with log-transform in sensitivity checks or dichotomized at 400 ng/mL in descriptions), first-line therapy category (curative, locoregional, systemic), hepatitis B and C status, ECOG performance status, the patient-specific mean SII in the exposure window, and the number of laboratory visits m. In extended models we added comorbidities and renal function.

### Data preprocessing and missing data

Laboratory values were inspected for implausible entries and gently winsorized at the 1 st and 99th percentiles within assay to reduce undue influence of extreme outliers. A non-winsorized analysis was conducted as a sensitivity check. Missing covariate and laboratory data were addressed using multiple imputation by chained equations with m = 20 datasets including all analysis variables, logarithm of survival time, and the event indicator. Imputed datasets were analyzed separately and estimates combined with Rubin’s rules. Because variability estimation requires at least three visits, patients with fewer than three visits were excluded a priori rather than imputed.

### Statistical analysis

Continuous variables were summarized as median (IQR) and categorical variables as counts (percent). Baseline characteristics were compared across quartiles of SII-VIM using Kruskal–Wallis tests for continuous variables and χ² tests for categorical variables. For OS, we fitted Cox proportional hazards models with SII-VIM as a continuous Z-score exposure and additionally as quartiles. Model 0 was unadjusted, Model 1 adjusted for age and sex, Model 2 (primary) additionally adjusted for BCLC stage, ALBI grade, AFP, first-line therapy, HBV/HCV status, ECOG, mean SII, and m, and Model 3 further added comorbidities and renal function. Proportional hazards assumptions were evaluated using scaled Schoenfeld residuals globally and for SII-VIM. Where indicated we included time-interaction terms, although no violations were detected in the primary model.

#### Sample size and power

Prior to data extraction we used Schoenfeld’s approximation for a continuous predictor to estimate that detecting a hazard ratio of 1.20–1.25 per 1-SD increase in SII-VIM with two-sided α = 0.05 and 80% power would require approximately 180–240 deaths, implying a target cohort of roughly 450–600 patients given expected event rates in our setting. The realized cohort and event counts met these targets.

#### Internal validation and rationale for no split‑sample CV

Model performance was assessed using bootstrap optimism correction with 1,000 resamples for Harrell’s C‑index, time‑dependent AUC at prespecified horizons, and the calibration slope, 95% CIs were obtained by bootstrap. Given the event count, a train/test split or k‑fold cross‑validation would reduce effective information and increase variance without providing external transportability.

#### Decision curve analysis (DCA)

To evaluate clinical utility, we computed time‑dependent net benefit at 12 months using the IPCW/Kaplan–Meier formulation of DCA for censored outcomes, comparing a baseline clinical model (BCLC stage, ALBI grade, AFP, first‑line therapy, age, sex, HBV/HCV, ECOG) with the same model plus SIIVIM. Predicted 12‑month risk was obtained from the Cox model (baseline survival × linear predictor). We examined risk thresholds from 10% to 35% (pre‑specified as plausible decision ranges for intensified surveillance/supportive measures), generated 1,000 bootstrap percentile confidence bands, and reported net benefit and net reduction in unnecessary interventions per 100 patients.

#### Therapy granularity and toxicity‑guarded sensitivities

Because treatment can acutely perturb inflammatory indices, we conducted three prespecified sensitivities. (1) Granular therapy adjustment: we replaced the broad first‑line therapy category with resection vs. ablation, TACE vs. HAIC vs. RT, and TKI vs. IO‑based (IO alone or IO + TKI) indicators. (2) Toxicity‑guarded exposure windows: we recomputed SIIVIM excluding ± 7 days around curative/locoregional procedures and excluding days 0–14 after initiation of systemic therapy, retaining patients with ≥ 3 remaining visits; the analysis used the same covariates as Model 2 (omitting m if constant). (3) Residualized variability: we regressed SIIVIM on the patient‑specific mean SII, m, and the granular therapy indicators (ordinary least squares) and used the studentized residuals (Z‑standardized) as the exposure. Each model used the day‑90 landmark Cox specification and Model 2 covariates.

All analyses were performed in R (Version 4.4). Two-sided p-values < 0.05 were considered statistically significant unless otherwise specified for FDR control, and confidence intervals were computed at the 95% level.

## Result

### Cohort assembly and baseline characteristics

**Fig. 1:**
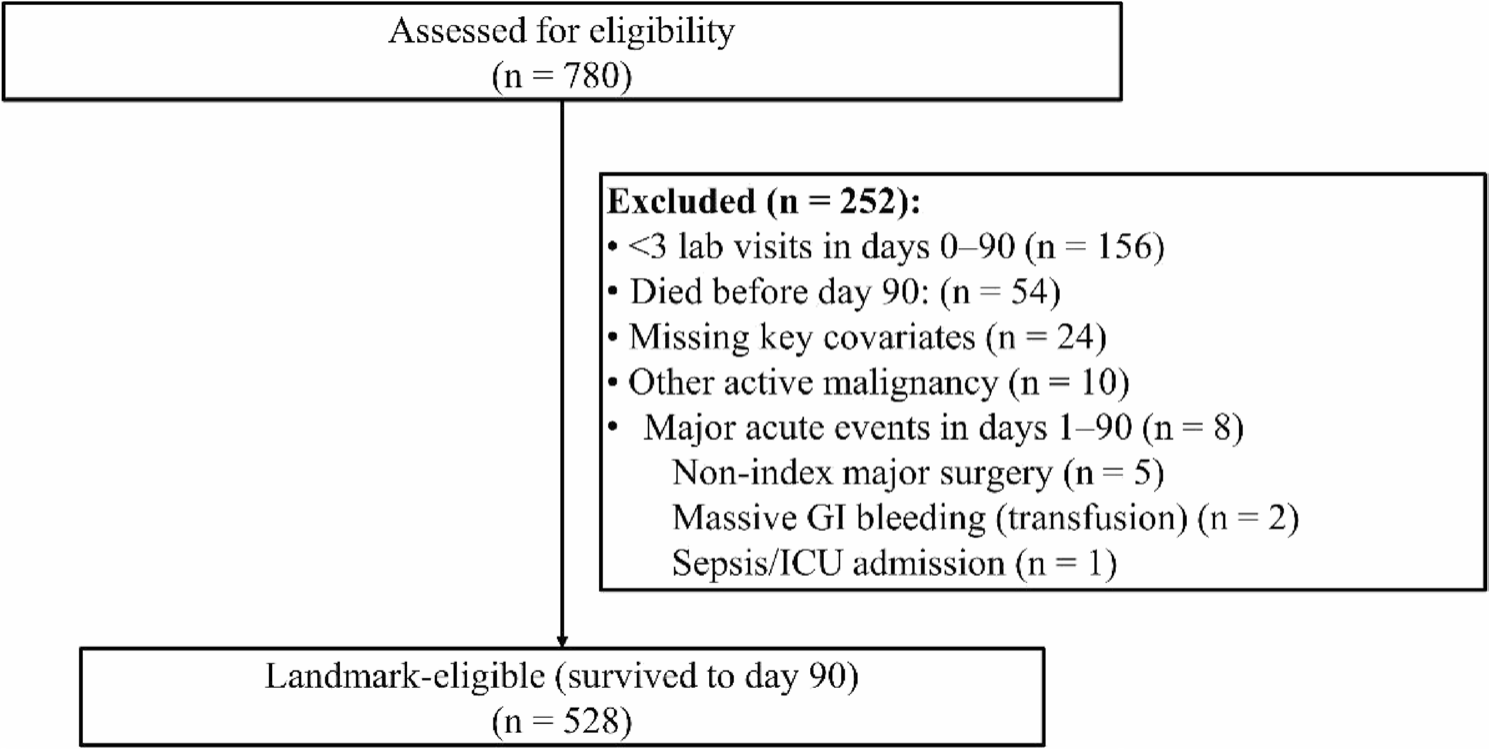
Flow of patients screened, exclusions, and the final landmark‑eligible cohort who survived to day 90 and entered time‑to‑event analyses. “Visit” denotes a same‑day lab panel; SII‑VIM (SIIVIM) and other visit‑to‑visit variability (VVV) metrics were computed from all labs in days 0–90 before the landmark. Primary outcome was overall survival from day 90. Key exclusions included < 3 visits in days 0–90 (*n* = 156) and death before day 90 (*n* = 54). This design mitigates immortal‑time bias by fixing the start of follow‑up at day 90. Abbreviations: HCC, hepatocellular carcinoma; VVV, visit‑to‑visit variability

From 780 patients screened between June 2020 and December 2024, 252 were excluded. Most commonly due to fewer than three laboratory visits within days 0–90 (*n* = 156) or death before day 90 (*n* = 54), yielding 528 landmark-eligible patients for analysis (Fig. 1). Median follow-up from the day-90 landmark was 25.1 months, during which 216 deaths occurred (41.0%). The median number of laboratory visits in days 0–90 was 4 (IQR 3–6), with 29.9% having three, 25.9% four, 18.6% five, 12.9% six, and 12.7% seven or more visits. Baseline features are summarized in Table 1. Median age was 58 years, 81.1% were male, 66.0% had HBV, and BCLC stages were A/B/C in 26.0%/44.0%/30.1%. Across quartiles of SII-VIM, higher variability coincided with more advanced tumor burden and worse liver function—BCLC distribution (*p* < 0.001), macrovascular invasion (26.3% overall; *p* < 0.001), extrahepatic metastasis (18.0% overall; *p* < 0.001), AFP >400 ng/mL (38.1%; *p* < 0.001), and ALBI grade (*p* = 0.009)—and a lower prevalence of ECOG 0 (*p* = 0.048), while age, sex, HBV/HCV, and cirrhosis were similar. Mean SII increased stepwise across variability quartiles (*p* < 0.001), and the number of visits showed a small gradient (*p* = 0.041), both of which were prespecified adjusters in multivariable models (Table 1). Using the prespecified operational cutoff, high variability corresponded to SII‑VIM ≥ [Q3–Q4 boundary] on the raw scale. To further characterize visit opportunity, we tabulated laboratory visits within days 0–90 by SIIVIM quartiles (Supplementary Table S1). Visit frequency showed only modest differences across variability quartiles, with a higher proportion of ≥ 7 visits in Q4 and similar medians otherwise (overall median 4 [IQR 3–6]; Q1 4 [3,–5], Q2 4 [3,–6], Q3 4 [3,–6], Q4 5 [3,–7]; global *p* = 0.041). Baseline (day‑0) cell counts are consistent with the variability gradient (Table 1 and Supplementary Table S2): neutrophils and platelets were higher and lymphocytes lower in higher‑variability quartiles (all *p* < 0.001 by Kruskal–Wallis).

**Table 1:** Baseline characteristics overall and by SII‑VIM quartiles

Characteristic	Overall (*N* = 528)	Q1 (lowest, *n* = 132)	Q2 (*n* = 132)	Q3 (*n* = 132)	Q4 (highest, *n* = 132)	*p*-value
Age (years), median (IQR)	58 (50–66)	58 (50–66)	57 (49–66)	58 (50–67)	59 (51–67)	0.232
Male sex, n (%)	428 (81.1)	104 (78.8)	106 (80.3)	109 (82.6)	109 (82.6)	0.584
HBV positive, n (%)	348 (66.0)	84 (63.6)	85 (64.4)	89 (67.4)	90 (68.2)	0.705
HCV positive, n (%)	57 (10.8)	16 (12.1)	15 (11.4)	14 (10.6)	12 (9.1)	0.822
Cirrhosis, n (%)	380 (72.0)	90 (68.2)	92 (69.7)	98 (74.2)	100 (75.8)	0.207
BCLC stage, n (%)						< 0.001
**• **A	137 (26.0)	54 (40.9)	38 (28.8)	25 (18.9)	20 (15.2)	
**• **B	232 (44.0)	58 (43.9)	62 (47.0)	59 (44.7)	53 (40.2)	
**• **C	159 (30.1)	20 (15.2)	32 (24.2)	48 (36.4)	59 (44.7)	
ALBI grade — n (%)						0.009
**•** 1	164 (31.1)	52 (39.4)	43 (32.6)	36 (27.3)	33 (25.0)	
**•** 2	275 (52.1)	64 (48.5)	69 (52.3)	69 (52.3)	73 (55.3)	
**•** 3	89 (16.9)	16 (12.1)	20 (15.2)	27 (20.5)	26 (19.7)	
Macrovascular invasion, n (%)	139 (26.3)	20 (15.2)	28 (21.2)	42 (31.8)	49 (37.1)	< 0.001
Extrahepatic metastasis, n (%)	95 (18.0)	12 (9.1)	18 (13.6)	28 (21.2)	37 (28.0)	< 0.001
AFP > 400 ng/mL, n (%)	201 (38.1)	35 (26.5)	43 (32.6)	58 (43.9)	65 (49.2)	< 0.001
First-line therapy, n (%)						< 0.001
**• **Curative (resection/ablation)	174 (33.0)	58 (43.9)	48 (36.4)	35 (26.5)	33 (25.0)	
**• **Locoregional (TACE/HAIC/RT)	222 (42.0)	54 (40.9)	56 (42.4)	56 (42.4)	56 (42.4)	
**•** Systemic (TKI/IO ± others)	132 (25.0)	20 (15.2)	28 (21.2)	41 (31.1)	43 (32.6)	
ECOG 0, n (%)	281 (53.2)	78 (59.1)	73 (55.3)	68 (51.5)	62 (47.0)	0.048
Mean SII during 0–90 d, median (IQR)	570 (350–820)	250 (190–310)	420 (360–490)	640 (570–710)	980 (870–1120)	< 0.001
Platelets at baseline (×10⁹/L), median (IQR)	215 (170–260)	180 (150–210)	210 (180–240)	240 (200–260)	260 (220–300)	< 0.001
Neutrophils at baseline (×10⁹/L), median (IQR)	3.3 (2.6–4.2)	2.5 (2.0–3.0)	3.0 (2.5–3.5)	3.6 (3.2–4.1)	4.5 (3.8–5.3)	< 0.001
Lymphocytes at baseline (×10⁹/L), median (IQR)	1.4 (1.1–1.8)	1.8 (1.5–2.1)	1.6 (1.3–1.8)	1.4 (1.2–1.6)	1.2 (1.0–1.4)	< 0.001
Lab visits m (0–90 d), median (IQR)	4 (3–6)	4 (3–5)	4 (3–6)	4 (3–6)	5 (3–7)	0.041

*SIIVIM quartile cut-points (raw scale*,* prior to Z-standardization)*: Q1 ≤ [Q1–Q2 boundary]; Q2: ([Q1–Q2] – [Q2–Q3]); Q3: ([Q2–Q3] – [Q3–Q4]); Q4 ≥ [Q3–Q4] (operational “high variability” cutoff)

Definition: SIIVIM = 100 × SD/mean^β using all SII values in days 0–90; cohort-wide β = 0.94 (95% CI 0.90–0.99)

Quartiles were formed on the *raw* SIIVIM distribution; modeling used Z-standardized SIIVIM

### Primary association with overall survival

In unadjusted analysis, each 1-SD increase in SII-VIM was associated with higher mortality (HR 1.34, 95% CI 1.22–1.48; *p* < 0.001). After adjusting for age, sex, BCLC stage, ALBI grade, AFP, first-line therapy, HBV/HCV status, ECOG status, the mean SII within days 0–90, and the number of laboratory visits, the association remained significant (Model 2 h 1.26, 95% CI 1.12–1.41; *p* < 0.001; FDR q = 0.001), and was similar in a further-adjusted model including comorbidities and renal function (HR 1.24, 95% CI 1.10–1.39; *p* < 0.001) (Table 2). When modeled categorically, the highest quartile of SII-VIM conferred a 72% higher hazard of death compared with the lowest quartile (HR 1.72, 95% CI 1.25–2.36; *p* = 0.001; FDR q = 0.004), with a significant trend across quartiles (p-trend < 0.001; FDR q = 0.002). In Model 2, the mean SII retained a weaker but significant association (HR 1.11 per 1 SD, 95% CI 1.01–1.22; *p* = 0.035; FDR q = 0.070), whereas the number of visits was not significant (HR 1.03 per visit, 95% CI 0.99–1.07; *p* = 0.13) (Table 2).

**Table 2:** Association of SII-VIM with overall survival (OS) from day-90 landmark

Exposure/Contrast	Model 0 h (95% CI)	*p*-value	Model 1 h (95% CI)	*p*-value	Model 2 h (95% CI)	*p*-value	q(FDR)*	Model 3 h (95% CI)	*p*-value
SII-VIM (per 1 SD)	1.34 (1.22–1.48)	< 0.001	1.33 (1.20–1.47)	< 0.001	1.26 (1.12–1.41)	< 0.001	0.001	1.24 (1.10–1.39)	< 0.001
Quartiles (Model 2)									
**• **Q1 (lowest)	Reference	—	—	—	Reference	—	—	Reference	—
**• **Q2 vs. Q1	—	—	—	—	1.12 (0.86–1.46)	0.392	0.48	1.10 (0.85–1.43)	0.481
**•** Q3 vs. Q1	—	—	—	—	1.29 (0.99–1.68)	0.059	0.09	1.26 (0.97–1.64)	0.085
**• **Q4 vs. Q1	—	—	—	—	1.72 (1.25–2.36)	0.001	0.004	1.66 (1.20–2.30)	0.002
p-trend across quartiles (Model 2)	—	—	—	—	< 0.001	—	0.002	—	—

### Dose–response and assumption checks

**Fig. 2:**
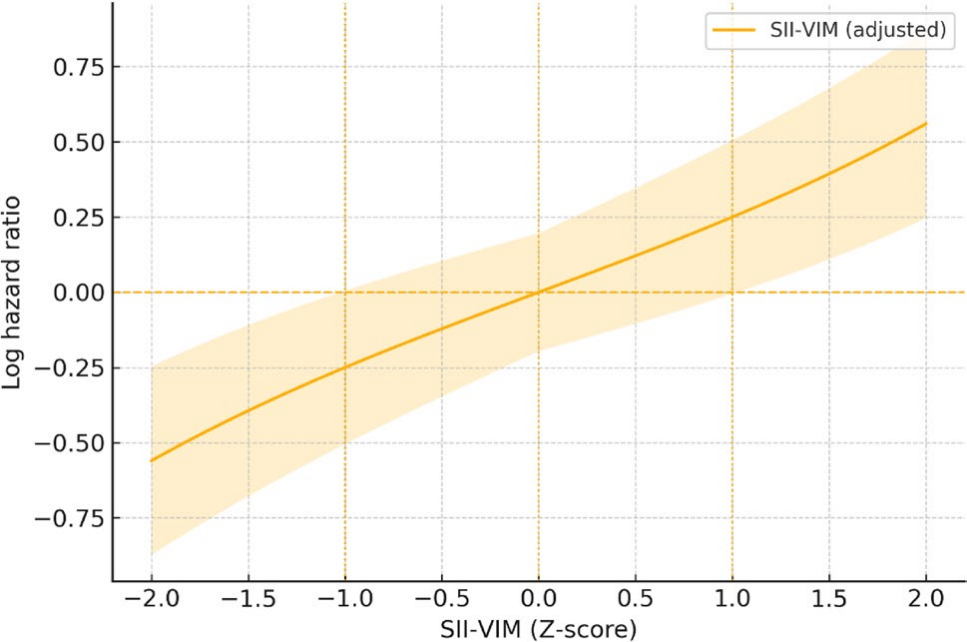
Dose–response of SII‑VIM and mortality. Adjusted relationship between SII‑VIM (Z‑score) in days 0–90 and the risk of death from the day‑90 landmark. The x‑axis is SII‑VIM in SD units (0 = cohort‑average variability; +1 SD ≈ notably more instability). The y‑axis shows the estimated log‑hazard (or plot as HR if preferred) with 95% CIs. Model adjusts for age, sex, BCLC, ALBI, AFP, first‑line therapy class, HBV/HCV, ECOG, the patient‑specific mean SII, and number of visits (m); proportional hazards assumptions were satisfied. Abbreviations: SII‑VIM, variability independent of the mean for SII

Restricted cubic splines demonstrated a monotonic, approximately linear increase in log-hazard with higher SII-VIM, with adjusted HRs of 1.12 at + 0.5 SD (95% CI 1.04–1.21), 1.28 at + 1.0 SD (95% CI 1.13–1.45), and 1.74 at + 2.0 SD (95% CI 1.33–2.26), and lower risk at negative Z-scores (Fig. 2). Proportional hazards assumptions were not violated for the global model (*p* = 0.62) or for SII-VIM specifically (*p* = 0.44) in Model 2 (Table 2).

### Incremental predictive performance

**Fig. 3:**
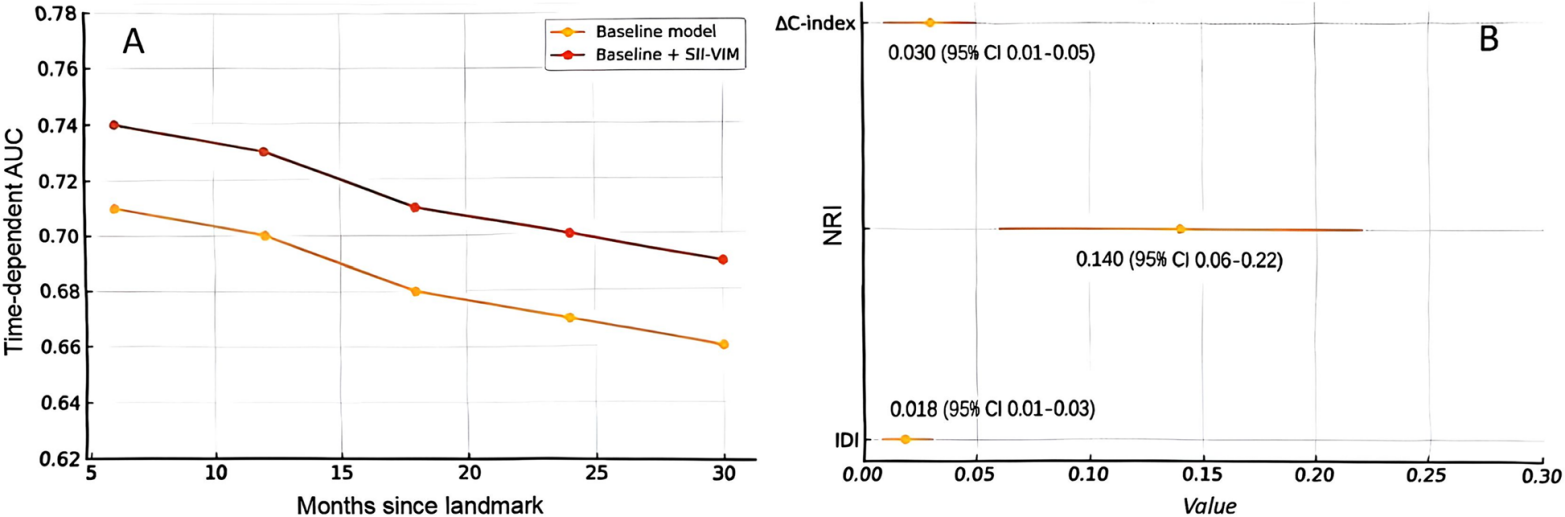
**A** Time‑dependent AUC by horizon: baseline model vs. + SII‑VIM. Discrimination over time (6–30 months) from the day‑90 landmark comparing a baseline clinical model (BCLC, ALBI, AFP, therapy class, and covariates) with the same model plus SII‑VIM. Points/lines are time‑dependent AUCs with bootstrap 95% CIs (1,000 resamples). **B** Global performance and calibration: baseline vs. + SII‑VIM. Summary of model performance at the day‑90 landmark. C‑index improves from 0.68 to 0.71 (Δ = 0.03; 95% CI 0.01–0.05). Reclassification at 12 months improves (continuous NRI = 0.14, IDI = 0.018), indicating more accurate risk ranking and probabilities

Adding SII-VIM to a baseline clinical model comprising BCLC stage, ALBI grade, AFP, first-line therapy, and covariates improved global discrimination, increasing Harrell’s C-index from 0.68 to 0.71 (Δ = 0.03; 95% CI 0.01–0.05). Time-dependent AUCs increased by about 0.03 at each prespecified time point, from 0.71 to 0.74 at 6 months, 0.70 to 0.73 at 12 months, 0.68 to 0.71 at 18 months, 0.67 to 0.70 at 24 months, and 0.66 to 0.69 at 30 months (Fig. 3A). Reclassification and discrimination gains were supported by a continuous NRI of 0.14 (95% CI 0.06–0.22) and an IDI of 0.018 (95% CI 0.008–0.030), with good calibration of the augmented model (bootstrap optimism-corrected slope 0.98) (Fig. 3B).

Across clinically relevant thresholds (10–30%), the + SIIVIM model demonstrated higher net benefit than the baseline model and the treat‑all strategy (Supplementary Figure S1). For example, at a 20% risk threshold the net benefit increased by 0.019 (95% CI 0.006–0.031), corresponding to ≈ 8 fewer unnecessary interventions per 100 patients while maintaining similar sensitivity for events. Gains were smaller at higher thresholds (e.g., ΔNB ≈ 0.015 at 30%) and attenuated near ≥ 35%, where curves converged. These findings indicate potential clinical utility of SIIVIM‑augmented stratification beyond improvements in C‑index, AUC, and NRI/IDI (Supplementary Table S1).

### Secondary and robustness analyses

Results were consistent across alternative variability metrics and exposure definitions (Table 3). The association of SII-ARV with OS was significant (HR 1.20 per 1 SD, 95% CI 1.08–1.34; *p* = 0.001; FDR q = 0.006). Recomputing the exposure over a 0–180-day window yielded an HR of 1.22 per 1 SD (95% CI 1.09–1.37; *p* < 0.001; FDR q = 0.004), and treating SII-VIM as a time-varying covariate updated every 90 days produced an HR of 1.18 (95% CI 1.07–1.30; *p* = 0.001; FDR q = 0.006). Across other indices, NLR-VIM showed a weaker but significant association (HR 1.15, 95% CI 1.05–1.26; *p* = 0.003; FDR q = 0.015), whereas PLR-VIM was not significant after multiplicity correction (HR 1.09, 95% CI 0.99–1.21; *p* = 0.078; FDR q = 0.156); LMR-VIM was neutral (HR 1.06, 95% CI 0.96–1.17; *p* = 0.230; FDR q = 0.310), SIRI-VIM was borderline (HR 1.10, 95% CI 0.99–1.22; *p* = 0.070; FDR q = 0.148), PNI-VIM trended oppositely (HR 0.94, 95% CI 0.85–1.04; *p* = 0.230; FDR q = 0.310), and ALBI variability was not significant (HR 1.08, 95% CI 0.97–1.20; *p* = 0.160; FDR q = 0.240) (Table 3).

**Table 3:** Secondary indices and robustness analyses (adjusted; multiplicity-controlled)

Exposure/Analysis	Metric/Contrast	Adjusted HR (95% CI)	*p*-value	q (FDR)
SII-ARV	Per 1 SD	1.20 (1.08–1.34)	0.001	0.006
SII-VIM	Per 1 SD; 0–180 d window	1.22 (1.09–1.37)	< 0.001	0.004
SII-VIM	Time-varying (rolling)	1.18 (1.07–1.30)	0.001	0.006
NLR-VIM	Per 1 SD	1.15 (1.05–1.26)	0.003	0.015
PLR-VIM	Per 1 SD	1.09 (0.99–1.21)	0.078	0.156
LMR-VIM	Per 1 SD	1.06 (0.96–1.17)	0.230	0.310
SIRI-VIM	Per 1 SD	1.10 (0.99–1.22)	0.070	0.148
PNI-VIM	Per 1 SD	0.94 (0.85–1.04)	0.230	0.310
ALBI variability (VIM)	Per 1 SD	1.08 (0.97–1.20)	0.160	0.240
RFS (curative subset, *n* = 174)	SII-VIM per 1 SD	1.22 (1.01–1.48)	0.040	0.080
PFS (non-curative subset, *n* = 354)	SII-VIM per 1 SD	1.19 (1.06–1.33)	0.003	0.012
Subgroup interaction: BCLC (A/B vs. C)	Interaction p	—	0.282	—
Subgroup interaction: HBV (pos vs. neg)	Interaction p	—	0.344	—
Subgroup interaction: therapy (curative vs. non-cur.)	Interaction p	—	0.121	—

The cohort‑wide scaling exponent for the SII mean–variance relationship was β = 0.94 (95% CI 0.90–0.99; R^2^ = 0.62). Allowing β to vary by BCLC stage did not improve fit (heterogeneity *p* = 0.31), with similar stage‑specific estimates (A 0.91 [0.83–1.00]; B 0.94 [0.88–1.00]; C 0.95 [0.89–1.01]). Patterns were comparable across therapy categories (heterogeneity *p* = 0.27) and ALBI grades (*p* = 0.21) (Supplementary Table S3). Recomputing SIIVIM using subgroup‑specific β values (β_g) yielded effect sizes nearly identical to the primary model (BCLC‑specific β_g: HR 1.25, 95% CI 1.11–1.41; therapy‑specific β_g: HR 1.25, 95% CI 1.11–1.40; ALBI‑specific β_g: HR 1.24, 95% CI 1.10–1.40; all *p* < 0.001), and these alternative exposures were highly correlated with SIIVIM computed using the pooled β (*r* ≥ 0.98; Supplementary Table S4). Collectively, these findings suggest that using a single cohort‑wide β for VIM does not mask materially different mean–variance relationships across clinically defined HCC subgroups.

Refining first‑line therapy into resection/ablation, TACE/HAIC/RT, and TKI/IO‑based classes did not materially change the association of SIIVIM with OS (HR 1.25; 95% CI 1.11–1.41; *p* < 0.001). Excluding ± 7 days around procedures (curative/locoregional) and days 0–14 after systemic‑therapy initiation yielded a similar estimate (HR 1.23; 95% CI 1.09–1.39; *p* = 0.001), with ≥ 3 remaining visits preserved in most patients. Using residualized SIIVIM orthogonal to mean SII, visit frequency, and granular therapy produced HR 1.22; 95% CI 1.08–1.38; *p* = 0.001. Therapy‑stratified interaction remained nonsignificant (interaction *p* = 0.12), arguing against treatment toxicity as the sole driver of the variability signal (Supplementary Table S5).

We examined whether the SIIVIM–OS association could be driven by visit intensity (Supplementary Table S6). When SIIVIM was recomputed from the first three visits only (fixed‑k), the association was similar to the primary estimate (HR 1.24; 95% CI 1.10–1.40; *p* = 0.001). Restricting the cohort to patients with exactly three visits yielded HR 1.25 (95% CI 1.04–1.50; *p* = 0.017). Modeling m flexibly with restricted cubic splines or using log(m) produced HRs of 1.25 (95% CI 1.11–1.41; *p* < 0.001). After applying stabilized visit‑intensity IPW, the association remained (HR 1.24; 95% CI 1.09–1.41; *p* = 0.001). These findings, together with manuscript‑reported analyses using a 0–180 d window (HR 1.22) and time‑varying SIIVIM (HR 1.18), suggest that the SIIVIM signal is not explained by testing frequency alone.

### Subgroups and secondary endpoints

There was no evidence of effect modification by BCLC stage (interaction *p* = 0.28) or HBV status (interaction *p* = 0.34), and only a nonsignificant trend toward stronger associations in non-curative versus curative therapy settings (interaction *p* = 0.12) (Table 3). In endpoint-specific analyses, higher SII-VIM was associated with recurrence-free survival in the curative subset (HR 1.22 per 1 SD, 95% CI 1.01–1.48; *p* = 0.040; FDR q = 0.080) using a Fine–Gray model and with progression-free survival in the non-curative subset (HR 1.19 per 1 SD, 95% CI 1.06–1.33; *p* = 0.003; FDR q = 0.012) using a counting-process Cox model, supporting the robustness of the primary OS findings (Table 3).

## Discussion

In this landmarked HCC cohort, higher early variability in the systemic immune-inflammation index independent of the mean (SII-VIM) was independently associated with mortality and provided incremental prognostic information beyond BCLC stage, ALBI grade, AFP, first-line therapy, HBV/HCV status, ECOG performance status, the patient-specific mean SII, and testing frequency. In the fully adjusted model, each 1-SD increase in SII-VIM conferred a 26% higher hazard of death, and patients in the highest versus lowest SII-VIM quartile had a 72% higher risk. The exposure–response was monotonic on restricted cubic splines and satisfied proportional hazards assumptions. Findings were robust across alternative variability metrics and specifications, including SII-ARV (HR 1.20 per 1 SD, 95% CI 1.08–1.34), an expanded 0–180-day exposure window (HR 1.22, 95% CI 1.09–1.37), and a time-varying analysis updating VVV every 90 days (HR 1.18, 95% CI 1.07–1.30), with no significant effect modification by BCLC stage or HBV status and consistent signals across treatment strata.

In our landmarked cohort, higher early variability in the systemic immune-inflammation index was associated with worse OS with a monotonic dose–response, a pattern plausibly reflecting an unstable inflammatory–immune equilibrium that both fuels tumor biology and compromises treatment tolerance. At the tumor–immune interface, neutrophil extracellular traps can up-regulate CD73 via Notch2–NF-κB signaling and promote regulatory T-cell infiltration, facilitating immune escape in HCC [21]. Platelets potentiate epithelial–mesenchymal transition and metastasis in HCC through TGF-β1–induced autophagy, and platelet releasates enhance HCC proliferation, aligning with the platelet–neutrophil–lymphocyte architecture embedded in SII [22]. Concurrently, lymphocyte-mediated antitumor control is blunted by exhausted PD-1^high^ CD8^+^ T-cell programs that correlate with poor outcomes in HCC [23]. Beyond the tumor, cytokine volatility in cirrhosis—particularly IL-6—predicts decompensation and mortality, providing a mechanistic substrate for short-term spikes in peripheral inflammatory indices [24]. Intercurrent infections such as spontaneous bacterial peritonitis remain frequent and lethal precipitants in cirrhosis with or without HCC, adding further bursts of inflammation that could inflate visit-to-visit variability [25]. Finally, peri-treatment inflammatory perturbations around TACE and surgery track adverse outcomes in HCC, supporting the clinical salience of short-horizon fluctuations rather than single measurements [26].

Prior HCC studies and meta-analyses established that baseline SII and NLR measured at a single time-point are associated with poorer OS and recurrence across treatment settings, but they do not interrogate whether variability itself carries independent signal [27, 28]. Our data extend this literature by showing that SII-VVV, quantified as variability independent of the mean, predicts mortality even after adjusting for mean SII, stage, liver function, therapy, and test frequency. This approach is methodologically consistent with robust precedents from non-oncology fields in which visit-to-visit variability predicts events, and where VIM is specifically advocated to decouple variability from average level [29, 30]. By coupling VIM with a day-90 landmark to mitigate immortal-time bias and by confirming a roughly linear risk gradient on splines, the present study addresses key analytical limitations that have constrained prior oncology reports centered on single snapshots.

Because SII-VVV is derived from routine differentials and platelet counts, it can be implemented alongside BCLC staging and ALBI-based liver reserve in guideline-concordant HCC care pathways (AASLD/EASL), where dynamic risk stratification is increasingly emphasized but variability measures are not yet incorporated [31, 32]. In our cohort, adding SII-VVV to a baseline clinical model improved discrimination, yielded meaningful reclassification, and showed near-ideal calibration, an effect size typical of additive biomarkers layered onto established model. These metrics are standard for censored data and align with best practice for calibration reporting [33, 34]. Clinically, quartiles or data-driven cutpoints could flag high-VVV patients for closer surveillance or targeted evaluation for occult infection/inflammation, and could support shared decision-making about therapy intensity and supportive care. Decision-curve analysis provides a principled framework to gauge net benefit across risk thresholds if implemented at scale [35, 36].

In our single-center HCC cohort, adding SII-VIM to a baseline clinical model improved discrimination and showed positive 12-month decision-curve net benefit across 10–30% thresholds with good calibration, features that lend themselves to point-of-care automation and trial design. In electronic records, SII-VIM can be computed automatically from routine CBC-differential in the 0–90-day pre-landmark window and surfaced as an absolute 12-month risk (with/without SII-VIM) and a “high-variability” flag using the cohort’s Q4 boundary, mirroring how MELD-3.0 is already embedded for cirrhosis care. Modern EMR standards (SMART-on-FHIR/CDS Hooks) support scheduled computation, thresholded alerts, and audit-ready logging [35, 37, 38]. In clinical trials, SII-VIM is best treated as a prognostic covariate, pre-specified for covariate adjustment and stratified randomization (high vs. non-high variability), with transparent reporting per TRIPOD and decision-analytic justification of thresholds. Such use is complementary to contemporary first-line HCC regimens and does not presume treatment-predictive effects [26, 35, 39, 40]. Finally, given consistent evidence that SII is prognostic in HCC, operationalizing its instability (SII-VIM) provides a low-cost, EMR-ready way to flag immune–inflammatory volatility for risk-aligned follow-up and prespecified trial analyses, while allowing local recalibration of β and cut-points [27]. 

Important limitations arise from the observational, single-center design and the possibility of residual confounding despite careful adjustment. Generalizability may be constrained by the etiologic mix and practice patterns of our setting (predominantly HBV), measurement heterogeneity across assay platforms and calendar periods may have introduced noise, and reliance on visit density raises concerns about indication bias and incomplete capture of unmeasured acute events or concomitant medications that could transiently perturb blood counts. Although multiple imputation (m = 20) reduced missing-data bias, imprecision remains possible, and external validation was not performed. Although we adjusted for m and mean SII, and found only a modest gradient in visit opportunity with no independent association of m with OS, residual confounding by care intensity is possible. We acknowledge the potential for indication bias whereby clinical instability could prompt more frequent testing and inflate VVV. Although we adjusted for m, demonstrated similar effects in m‑restricted and first‑three‑visits sensitivities, and observed that m itself was not associated with OS after adjustment, residual confounding by care intensity may persist. We did not perform a separate training/testing split or k‑fold cross‑validation. In our setting, such approaches would sacrifice precision with limited added value. Instead, we used bootstrap optimism‑correction for discrimination and calibration. We acknowledge that these constitute internal validation only and do not replace external validation. To translate visit-to-visit variability into practice, prospective, multi-center studies should evaluate external transportability across etiologies (HBV, HCV, NASH) and health systems, compare VIM with alternative variability constructs and dynamic modeling strategies, and test integration with radiomics, genomic/immune signatures, and early on-treatment response. Interventional trials and pragmatic care pathways are also warranted to assess whether strategies that stabilize inflammatory–immune variability—such as optimized infection surveillance and control, antiviral management, and supportive-care bundles—can improve outcomes and enhance the clinical utility of VVV-augmented risk stratification within EHR-embedded calculators.

Early visit-to-visit variability—particularly SII-VIM—is a low-cost, dynamic marker that augments current prognostication in HCC, supporting its evaluation for clinical implementation following prospective validation. Because SII-VIM derives from routine differentials and platelets, it is readily implementable and well suited to integration with established tools (BCLC, ALBI) to refine surveillance intensity and supportive-care planning. Prospective, multi-center studies should now test transportability across etiologies and health systems and evaluate whether VVV-guided care pathways improve outcomes, paving the way for EHR-embedded risk calculators that operationalize this dynamic signal in everyday practice.

## Supplementary Information

Supplementary Material 1.

## Data Availability

Data sets generated during the current study are available from the corresponding author on reasonable request.
