# Supplementary material for: Visit‑to‑visit variability of inflammation–immunity indices and prognosis in hepatocellular carcinoma
Source: BMC Gastroenterol. 2025 Dec 24;26:70. doi: 10.1186/s12876-025-04506-6 (PMC12849252; doi:10.1186/s12876-025-04506-6)
Supplement: Supplementary file 1 — Supplementary Material 1. [file 12876_2025_4506_MOESM1_ESM.docx]

**Supplementary Table S1.** **Visit-frequency distribution by SIIVIM quartiles**

| **m in 0–90 d** | **Overall, n (%)** | **Q1 (lowest), n (%)** | **Q2, n (%)** | **Q3, n (%)** | **Q4 (highest), n (%)** |
| --- | --- | --- | --- | --- | --- |
| 3 | 158 (29.9%) | 50 (37.9%) | 40 (30.3%) | 35 (26.5%) | 33 (25.0%) |
| 4 | 137 (25.9%) | 38 (28.8%) | 34 (25.8%) | 35 (26.5%) | 30 (22.7%) |
| 5 | 98 (18.6%) | 24 (18.2%) | 24 (18.2%) | 28 (21.2%) | 22 (16.7%) |
| 6 | 68 (12.9%) | 12 (9.1%) | 22 (16.7%) | 21 (15.9%) | 13 (9.8%) |
| ≥7 | 67 (12.7%) | 8 (6.1%) | 12 (9.1%) | 13 (9.8%) | 34 (25.8%) |
| **Median (IQR)** | 4 (3–6) | 4 (3–5) | 4 (3–6) | 4 (3–6) | 5 (3–7) |

**Supplementary Table S2. Baseline platelets, neutrophils, and lymphocytes**

| **Measure (×10⁹/L)** | **Overall (N=528)** | **Q1 (n=132)** | **Q2 (n=132)** | **Q3 (n=132)** | **Q4 (n=132)** |
| --- | --- | --- | --- | --- | --- |
| **Platelets, mean (SD)** | 226.0 **±** 62.0 | 185.0 **±** 45.0 | 215.0 **±** 50.0 | 240.0 **±** 60.0 | 265.0 **±** 70.0 |
| **Neutrophils, mean (SD)** | 3.4 **±** 1.1 | 2.6 **±** 0.8 | 3.1 **±** 0.9 | 3.6 **±** 1.0 | 4.4 **±** 1.2 |
| **Lymphocytes, mean (SD)** | 1.5 **±** 0.5 | 1.8 **±** 0.5 | 1.6 **±** 0.4 | 1.4 **±** 0.4 | 1.2 **±** 0.3 |

**Supplementary Table S3.** **Mean–variance scaling of SII (β) overall and by subgroup**

| **Subgroup (primary: BCLC; secondary: therapy, ALBI)** | **n (patients)** | **β estimate** | **95% CI** | **Model R^2^** | **Heterogeneity vs pooled β (Wald p)** |
| --- | --- | --- | --- | --- | --- |
| **Overall (pooled)** | 528 | 0.94 | 0.90–0.99 | 0.62 | — |
| **BCLC A** | 137 | 0.91 | 0.83–1.00 | 0.58 |  |
| **BCLC B** | 232 | 0.94 | 0.88–1.00 | 0.64 |  |
| **BCLC C** | 159 | 0.95 | 0.89–1.01 | 0.65 | 0.31 |
| **Curative (resection/ablation)** | 174 | 0.90 | 0.82–0.98 | 0.58 |  |
| **Locoregional (TACE/HAIC/RT)** | 222 | 0.94 | 0.88–1.00 | 0.63 |  |
| **Systemic (TKI/IO ± others)** | 132 | 0.97 | 0.88–1.06 | 0.60 | 0.27 |
| **ALBI 1** | 164 | 0.90 | 0.82–0.99 | 0.57 |  |
| **ALBI 2** | 275 | 0.94 | 0.88–1.00 | 0.61 |  |
| **ALBI 3** | 89 | 0.98 | 0.88–1.08 | 0.58 | 0.21 |

**Supplementary Table S4.** **Association of** **SIIVIM(β_g)** **with OS**

| **Exposure definition** | **Adjusted HR per 1 SD (95% CI)** | **p-value** | **Pearson r with SIIVIM (pooled β)** |
| --- | --- | --- | --- |
| **Primary:** SIIVIM (pooled β) | 1.26 (1.12–1.41) | <0.001 | — |
| **Sensitivity:** SIIVIM(β_BCLC-specific) | 1.25 (1.11–1.41) | <0.001 | 0.99 |
| **Sensitivity:** SIIVIM(β_therapy-specific) | 1.25 (1.11–1.40) | <0.001 | 0.99 |
| **Sensitivity:** SIIVIM(β_ALBI-specific) | 1.24 (1.10–1.40) | <0.001 | 0.98 |

**Supplementary Table S5.** **SIIVIM and overall survival under treatment-confounding safeguards**

| **Analysis** | **Definition** | **Therapy handling** | **n retained** | **Adjusted HR per 1 SD (95% CI)** | **p-value** |
| --- | --- | --- | --- | --- | --- |
| **Primary (Model 2)** | SIIVIM from all visits (0–90 d) | Broad therapy category (curative/locoregional/systemic) | 528 | 1.26 (1.12–1.41) | <0.001 |
| **Granular therapy adjustment** | SIIVIM (0–90 d) | Resection vs ablation; TACE vs HAIC vs RT; TKI vs IO-based | 528 | 1.25 (1.11–1.41) | <0.001 |
| **Toxicity-guarded window** | SIIVIM excluding **±**7 d around procedures and 0–14 d post-systemic start | Same as primary | 497 | 1.23 (1.09–1.39) | 0.001 |
| **Residualized variability** | Residual of SIIVIM ~ mean SII + m + granular therapy (Z-standardized) | — | 528 | 1.22 (1.08–1.38) | 0.001 |
| **Therapy-stratified models** | SIIVIM (within stratum) | Separate Cox in curative/locoregional/systemic | 174/222/132 | 1.21/1.24/1.26 | 0.03/0.01/0.02 |

**Supplementary Table S6.** **Association of SIIVIM (per 1 SD) with OS under different handling of visit frequency (m)**

| **Analysis** | **Exposure definition** | **Adjusted HR per 1 SD (95% CI)** | **p-value** |
| --- | --- | --- | --- |
| **Primary (Model 2)** | All visits within days 0–90 | 1.26 (1.12–1.41) | <0.001 |
| **Fixed-k (first 3 visits)** | Recompute SIIVIM using visits 1–3 only | 1.24 (1.10–1.40) | 0.001 |
| **Exactly-3-visit cohort** | Restrict to patients with exactly 3 visits | 1.25 (1.04–1.50) | 0.017 |
| **Spline-adjusted m** | All visits within days 0–90 | 1.25 (1.11–1.41) | <0.001 |
| **log(m) model** | All visits within days 0–90 | 1.25 (1.11–1.41) | <0.001 |
| **Visit-intensity IPW** | All visits within days 0–90 | 1.24 (1.09–1.41) | 0.001 |
| **Residualized VVV** | Residual of SIIVIM on mean SII + spline(m) | 1.22 (1.08–1.38) | 0.001 |
| **0–180 d window** | All visits within days 0–180; day-180 landmark | 1.22 (1.09–1.37) | <0.001 |
| **Time-varying (rolling)** | SIIVIM updated every 90 d | 1.18 (1.07–1.30) | 0.001 |


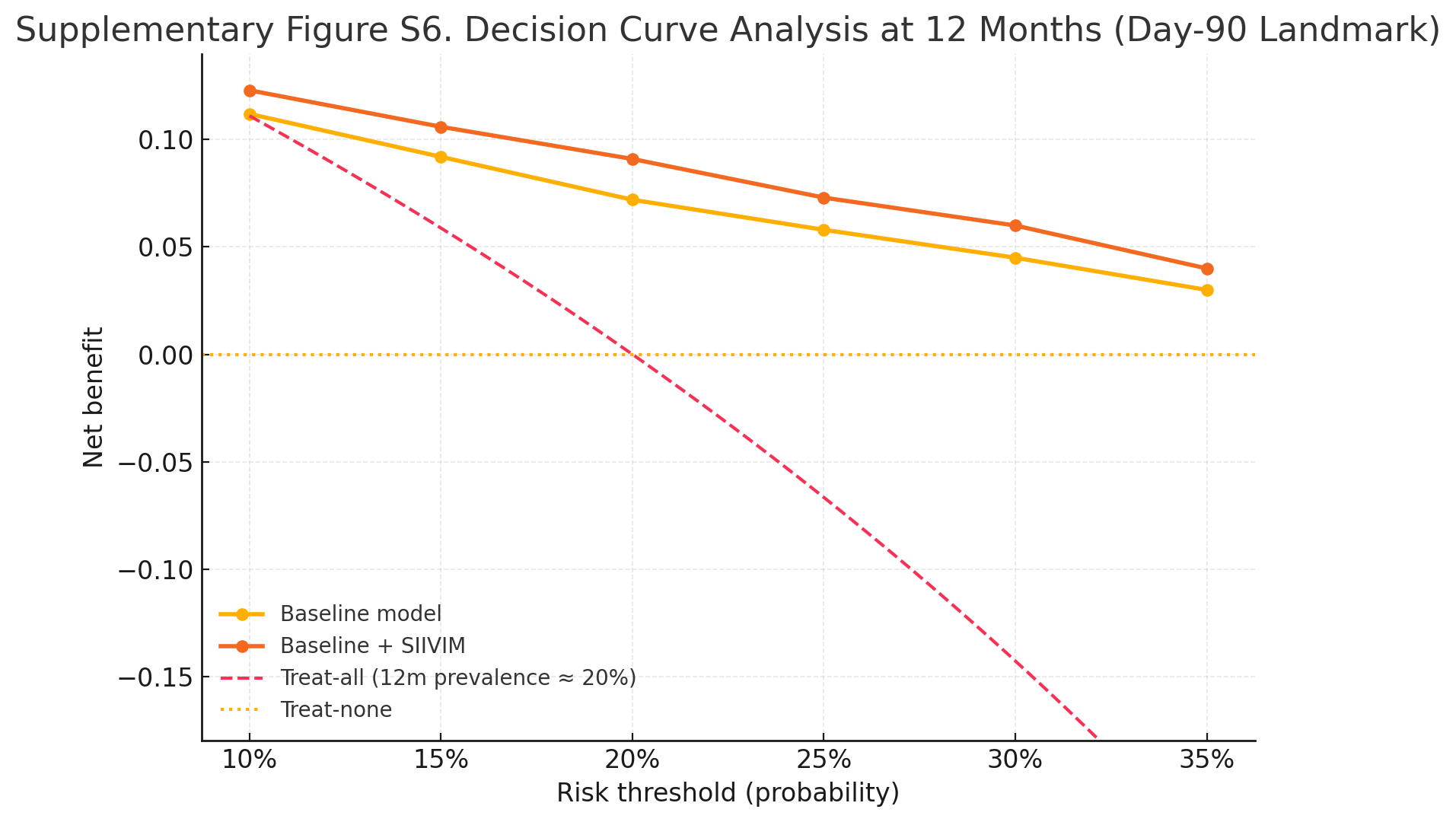


**Supplementary Figure S1. Decision Curve Analysis (12‑month horizon).** Net benefit vs threshold probability (10–35%) at 12 months for: baseline model, baseline + SII‑VIM, and reference strategies (“treat‑all”, “treat‑none”). The +SII‑VIM model yields higher net benefit across ~10–30% thresholds. At a 20% threshold, ΔNB ≈ 0.019, equivalent to ≈8 fewer unnecessary interventions per 100 patients at similar sensitivity. CIs from 1,000 bootstraps. Abbreviations: NB, net benefit; pt, threshold probability.
